# Venous thromboembolism at high altitude: mechanisms, risk stratification, and precision prevention

**DOI:** 10.3389/fphys.2026.1851375

**Published:** 2026-07-01

**Authors:** Jian Feng, Nan Zhang, Guan Yang, Xiao Wang, Juan Chen, Fuxiang Li, Ruiwu Dai, Hai Yi

**Affiliations:** 1 Department of Intensive Care Unit, The General Hospital of Western Theater Command, Chengdu, China; 2 Department of Hematology, The General Hospital of Western Theater Command, Sichuan Clinical Research Center for Hematological Disease, Branch of National Clinical Research Center for Hematological Disease, Chengdu, China; 3 Department of Pain Medicine, The General Hospital of Western Theater Command, Chengdu, China; 4 Department of General Surgery, The General Hospital of Western Theater Command, Chengdu, China

**Keywords:** high-altitude, hypoxia, pathophysiological mechanisms, precision prevention, risk assessment, venous thromboembolism

## Abstract

High-altitude environments constitute an increasingly important global health concern as exposure widens. This narrative review summarizes recent evidence on the epidemiology, pathophysiology, and clinical therapy of venous thromboembolism (VTE) in high-altitude environments. Multi-system crosstalk mediated by hypoxia-inducible factor (HIF)–based activation disrupts hemostatic homeostasis, inducing a distinctive high-altitude prothrombotic phenotype. Traditional VTE clinical guidelines, designed mainly for low-altitude populations, show considerable weaknesses in high-altitude conditions. A new three-dimensional (altitude–exposure–susceptibility) risk assessment model is urgently required. Through comparative assessment of the evidence, we discuss fundamental controversies in this area, the need to create multidimensional predictive models combined with altitude-specific physiological variables, and suggest specific management approaches for high-risk groups. Currently, significant gaps exist in epidemiological data collection, mechanistic characterization, and clinical trial evidence. Future priorities include multi-omics integration, novel animal models, and rigorously designed randomized controlled trials to enable a paradigm shift from population-based to precision prevention of high-altitude VTE. Target audience: clinicians, physiologists, and public health researchers.

## 1 Introduction

High-altitude environments are operationally defined as three tiers: high altitude (2500–3500 m; 8202–11,483 ft), very high altitude (3500–5500 m; 11,483–18,045 ft), and extreme altitude (>5500 m; >18,045 ft) (Hoiland et al., 2019; Sanou et al., 2023). These thresholds are applied consistently throughout this review. This review is intended primarily for clinicians managing patients in high-altitude settings—surgeons, intensivists, and emergency physicians—and secondarily for physiologists and public health researchers developing altitude-specific interventions.

With high-altitude activities increasingly common in both clinical and non-clinical settings, venous thromboembolism (VTE), deep vein thrombosis (DVT), and pulmonary embolism (PE) carry a significantly higher incidence at high altitude than at low altitude, placing them high on the public health agenda (Yang et al., 2022). The emerging literature has shown high altitude to be an autonomous risk factor for VTE, with a documented dose-response gradient (Yang et al., 2022). In orthopedic surgical patients at high altitude (≥2500 m), the probability of VTE is high (15 – 47%) after surgical procedure (Broggi et al., 2021; Jones et al., 2022; Plancher et al., 2025). The manifestation of high-altitude VTE frequently overlaps with altitude-related conditions such as high-altitude pulmonary edema (HAPE), generating significant diagnostic dilemmas; specifically, the risk of VTE is as high as 38.9% (hospitalized patients with severe HAPE) (Wu et al., 2022; Liu et al., 2023).

**Figure 1:**
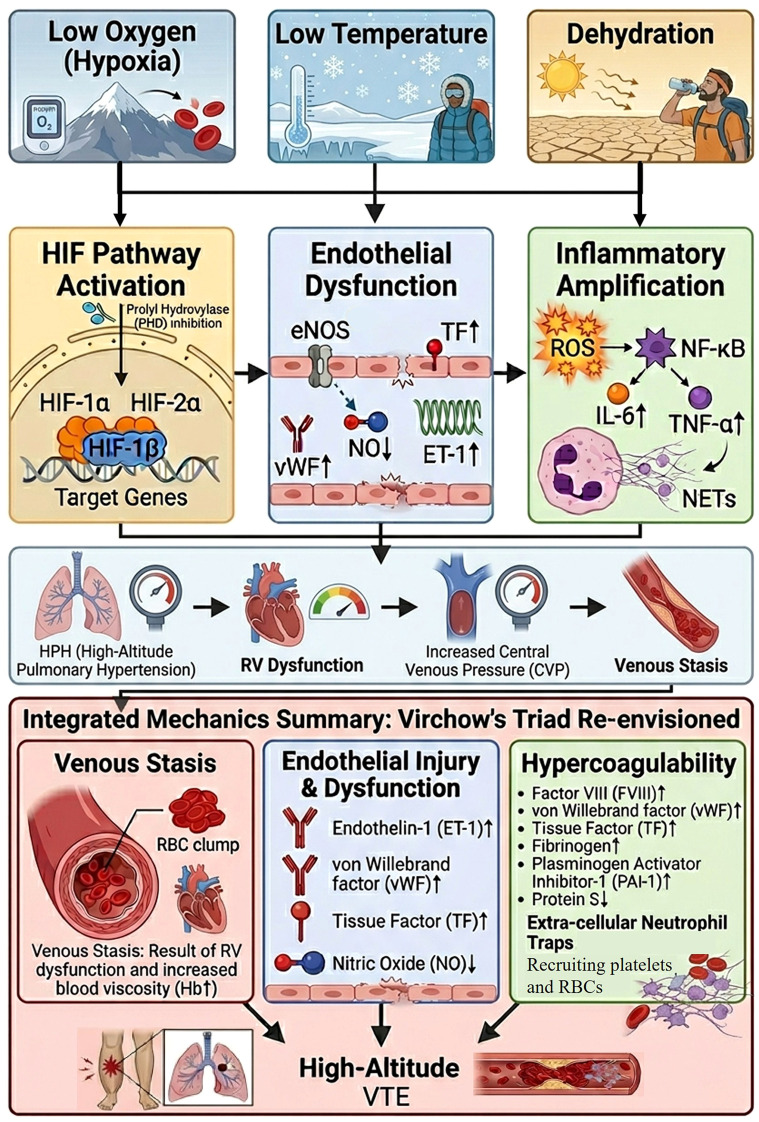
Integrated overview of key pathophysiological pathways and HIF-centered multi-stressor mechanistic model of high-altitude venous thromboembolism (VTE). High-altitude environmental stressors (hypoxia, low temperature, dehydration) converge on hypoxia-inducible factor (HIF) pathway activation, driving endothelial dysfunction (reduced nitric oxide [NO] bioavailability, elevated von Willebrand factor [vWF], tissue factor [TF], and endothelin-1 [ET-1]), inflammatory amplification (reactive oxygen species [ROS]–nuclear factor kappa-B [NF-κB]–interleukin-6 [IL-6]/tumor necrosis factor-alpha [TNF-α] cascade, neutrophil extracellular traps [NETs]), and hemodynamic deterioration (hypoxic pulmonary hypertension [HPH] → right ventricular [RV] dysfunction → increased central venous pressure [CVP] → venous stasis). The lower panel integrates these into Virchow triad components: venous stasis via secondary erythrocytosis and hyperviscosity; endothelial injury; and hypercoagulability (elevated factor VIII [FVIII], vWF, TF, fibrinogen, and plasminogen activator inhibitor-1 [PAI-1]; reduced protein S; NET-mediated platelet and erythrocyte recruitment).

Current VTE prevention and management strategies, based on sea-level clinical data, fail to adequately account for high-altitude physiological adaptations—such as HIF pathway activation, hemorheological alterations, and endothelial dysfunction (Tourn et al., 2025). Although existing evidence supports the combined use of the Caprini score and D-dimer testing for VTE risk prediction (Shi et al., 2025) and low molecular weight heparin (LMWH) as the first-line thromboprophylactic agent (Qiong et al., 2025), several critical research gaps remain: the lack of large-scale prospective cohort studies covering geographically diverse high-altitude regions, uncharacterized molecular targets within the hypoxia-coagulation-inflammation network, and insufficient safety and efficacy data for anticoagulants in special high-altitude populations (e.g., pregnant women, pediatric patients, and the elderly). This narrative review synthesizes the latest evidence spanning from the pathogenesis to clinical management of high-altitude VTE, and provides evidence-based guidance for precision-oriented prevention and treatment of VTE in high-altitude environments (**Figure 1**). This review advances beyond prior narrative reviews focused on air-travel thrombosis (Tourn et al., 2025) or postoperative orthopedic risk (Yang et al., 2022) by integrating the full altitude spectrum, proposing a mechanistic framework centered on HIF multi-stressor interactions, and addressing precision prevention for underrepresented populations including pregnant women, pediatric patients, and high-altitude natives.

As a narrative review, we systematically searched PubMed, Embase, and Web of Science from inception to May 2026 using terms: (‘high altitude’ OR ‘hypobaric hypoxia’) AND (‘venous thromboembolism’ OR ‘deep vein thrombosis’ OR ‘pulmonary embolism’). Reference lists were hand-searched. Non-English publications and grey literature were excluded, which may introduce selection bias.

## 2 Epidemiological characteristics of VTE in high-altitude environments

### 2.1 Altitude gradient and dose-response relationships

Meta-analyses of postoperative orthopedic cohorts demonstrate that the incidence of VTE is significantly higher in patients operated at high altitude than in low-altitude controls (Yang et al., 2022). This altitude-dependent risk gradient is particularly prominent in postoperative patients. For patients undergoing total knee arthroplasty (TKA) at high altitude (≥2500 m), the 30-day postoperative VTE risk is increased by 15 – 47% (in orthopedic surgical patients), and the 90-day risk by 20% (Plancher et al., 2025). In patients who underwent tibial plateau fracture surgery at high altitudes, the risks of DVT and PE within 90 days post-surgery are elevated by 21% and 27% (in tibial plateau fracture patients), respectively (Jones et al., 2022).

Exposure to extreme high altitudes exhibits a threshold effect on VTE risk, with a sharp increase in risk once the threshold is exceeded. Comparative studies between high and extreme high-altitude regions have found that patients who develop PE during the early phase of extreme altitude exposure have a markedly higher risk of concurrent lower-extremity DVT (OR 16.3; 95% CI, 1.2 – 223.2) (Xiong et al., 2024). The extremely wide confidence interval reflects small sample size and low statistical power; this association requires validation in large cohorts. A single-center study of PE patients at a mean altitude of 5041 m reported that this cohort was significantly younger and had markedly higher erythrocyte counts, hemoglobin levels, platelet counts, and uric acid levels (all p<0.05) compared with low-altitude controls (Wu et al., 2025). These findings point to unique pathophysiological characteristics of VTE at extreme altitudes. Published data on VTE risk at moderate altitudes (<2500 m) are limited and inconsistent; some small studies have reported no significant increase, although no large-scale negative study has been published to date.

### 2.2 Risk profiles across exposure patterns

The risk spectrum of VTE varies by exposure pattern and baseline susceptibility: short-term visitors, long-term migrants, and high-altitude natives exhibit distinct risk profiles. Populations with short-term high-altitude exposure (e.g., mountaineers and air travelers) can rapidly develop a hypercoagulable state even with brief hypoxic exposure (Jacobson et al., 2024). In healthy volunteers exposed to a simulated altitude of 5486 m for one hour, plasma von Willebrand factor (vWF) activity and D-dimer levels are substantially elevated, accompanied by shortened R and K times on thromboelastography (TEG) (Jacobson et al., 2024). Case reports (limited to 1–3 subjects) have described multi-site thrombotic events—including cerebral venous sinus thrombosis (CVST), PE, and right ventricular thrombosis—in young individuals within three days of rapid ascent to high altitudes (Gan et al., 2025), though these findings await further validation in populations with actual high-altitude exposure.

Long-term migrants and high-altitude natives are at risk of VTE due to chronic adaptive changes and cumulative hypoxic effects. Among hospitalized patients with acute exacerbation of chronic obstructive pulmonary disease (AECOPD) at high altitudes, the prevalence of PE is 29.6% (AECOPD patients); multivariate logistic regression analysis identified a Padua score >3 and D-dimer >1 mg/L as independent risk factors for PE (p<0.05) (Yang et al., 2024). Prospective studies conducted in Tibet reported an overall VTE incidence of 13.5% (hospitalized patients in Tibet); notably, the incidence rose to 42.9% (ICU patients without prophylaxis) in ICU patients who did not receive standardized thromboprophylaxis, compared with 35.9% in those who received LMWH prophylaxis (Qiong et al., 2025; Shi et al., 2025). Synergistic risk effects are particularly pronounced in specific clinical scenarios: the incidence of VTE is 38.9% (95% CI, 21.5 – 59.2) (hospitalized patients with severe HAPE) (Liu et al., 2023), and these patients present with more severe clinical manifestations and a high incidence of respiratory failure. This phenomenon reflects the synergistic effects of severe hypoxia, prolonged immobilization, and excessive inflammatory responses in this patient group.

### 2.3 Clinical characteristics and diagnostic challenges

The diagnosis of high-altitude VTE is complicated by a “symptom overlap syndrome”, whereby the classic clinical symptoms of PE (dyspnea and chest pain) closely mimic those of HAPE (Wu et al., 2022). In a small case series (n=7) of extreme high-altitude VTE, 6 patients had first been misdiagnosed with pulmonary inflammation and 4 with HAPE (Wu et al., 2022); the results cannot be generalized to the wider high-altitude population. The cardinal symptoms of CVST (headache and nausea) are comparable to acute mountain sickness (AMS) (Yang et al., 2023). Consequently, there is a prolonged period between the onset of symptoms and the definite diagnosis of patients with high-altitude VTE, especially CVST, in relation to patients with low-altitude VTE, which causes considerable diagnostic delays (Yang et al., 2023). The interpretation of laboratory test results is also another source of diagnostic problems. As an example, D-dimer is a major screening biomarker of VTE; however, hypoxia at high altitudes may result in a physiological increase in baseline levels of D-dimer, with the extent of increase also dependent on altitude and individual adaptation status (Jacobson et al., 2024). Among patients with severe HAPE, those who develop concurrent VTE have substantially higher D-dimer levels (median 7.81 mg/L vs. 2.90 mg/L) (Liu et al., 2023). Reliance on D-dimer testing alone may therefore lead to false positive results, highlighting the need for integration with clinical probability assessments. Key studies are summarized in **Table 1**.

**Table 1:** Summary of key studies on high-altitude venous thromboembolism.

Study (first author, year)	Population / setting	Altitude (m)	Design	Key findings
(Yang et al., 2022)	Postoperative orthopedic	≥2500	Meta-analysis	High altitude is an independent VTE risk factor with dose-response gradient
(Plancher et al., 2025)	TKA patients	≥1219	Database study	30- and 90-day VTE risk increased at high altitude
(Jones et al., 2022)	Tibial plateau fracture	High altitude	Retrospective	DVT and PE risks elevated 21% and 27% at 90 days
(Broggi et al., 2021)	Pelvic/acetabular trauma	High altitude	Retrospective	Higher altitude increases post-injury VTE risk
(Liu et al., 2023)	Severe HAPE patients	Plateau regions	Prospective	VTE incidence 38.9%; severe clinical manifestations
(Wu et al., 2022)	Extreme altitude PE	>5500	Case series (n=7)	High misdiagnosis rate; symptom overlap with HAPE
(Jacobson et al., 2024)	Healthy volunteers	5486 (simulated)	Experimental	vWF and D-dimer elevated after 1-hour hypoxia
(Shi et al., 2025)	Hospitalized patients	High altitude	Prospective validation	Combined Caprini-D-dimer model AUC 0.89
(Qiong et al., 2025)	ICU patients	Tibet	Prospective cohort	LMWH reduced VTE from 42.9% to 35.9%
(Xiong et al., 2024)	VTE patients	Different altitudes	Cross-sectional	Altitude-dependent VTE profiles
(Wu et al., 2025)	PE patients	5041 (mean)	Single-center retrospective	Younger age, higher hematocrit, shorter thrombus dissolution
(Gan et al., 2025)	Multi-site thrombosis	High altitude	Case report	Cerebral, pulmonary, and ventricular thrombosis in rapid ascender
(Yang et al., 2024)	AECOPD patients	Plateau regions	Prospective cohort	PE prevalence 29.6%; Padua score and D-dimer as risk factors
(Yang et al., 2023)	CVST patients	Plateau areas	Retrospective	Diagnostic delays due to AMS symptom overlap
(Srivastava et al., 2020)	VTE patients	High altitude	Gene expression	Ten genes upregulated (CDH18, FGA, EDNBR, GATA2, MAPK9)
(Arya et al., 2026)	VTE patients	High altitude	Epigenetic	Hypoxia-mediated methylation of coagulation-related genes
(Li et al., 2022)	Healthy volunteers	High altitude	Experimental	Hypoxia and low temperature upregulate transferrin
(Ninivaggi et al., 2024)	Healthy volunteers	High altitude	Experimental	Exercise and hypoxia-induced hypercoagulability counterbalanced in women
(Zhao et al., 2023)	TKA patients	High altitude	Retrospective	Altered coagulation and fibrinolysis parameters; bleeding risk
(Majumder and Nguyen, 2021)	General review	N/A	Review	Protein S function and regulation in coagulation
(Fan et al., 2020)	Healthy volunteers	Different altitudes	Cross-sectional	Altitude-dependent vascular endothelial dysfunction
(Tremblay et al., 2020)	Healthy volunteers	Hypobaric hypoxia	Experimental	Endothelial function and shear stress time course
(Yu et al., 2020)	Animal model	N/A	Experimental (animal)	AMPK activation inhibits tissue factor-triggered intestinal ischemia
(Nicolai et al., 2020)	Inflammatory model	N/A	Experimental (animal)	Platelet haptotaxis in inflammation and infection
(Imiela et al., 2024)	Animal models	N/A	Review	Acute PE and immunity
(Schulman et al., 2024)	General review	N/A	Review	Basic principles of venous thrombosis pathophysiology
(Diep and Garcia, 2020)	General review	N/A	Review	Aspirin for VTE prophylaxis: limited evidence
(Luks and Grissom, 2021)	Post-COVID patients	High altitude	Review	Return to high altitude after COVID-19 recovery
(Nimmatoori et al., 2020)	VTE patient	High altitude	Case report	Rapidly progressing multifocal high-altitude vascular thrombosis
(Wisniewska et al., 2020)	Athletes	Moderate altitude	Prospective	Erythropoietin and VEGF changes after altitude training
(Cerda-Kohler et al., 2022)	Elite rowers	Moderate altitude	Prospective	Improved submaximal but not maximal performance
(Furuto et al., 2020)	CKD patients	Travel settings	Review	Health risks of high-altitude travel for CKD patients
(Quan et al., 2023)	Post-COVID patients	High altitude	Review	Cognitive dysfunction in high-risk populations

N/A indicates not applicable (review, animal model, or general population study without specific altitude restriction).

### 2.4 Extreme altitude (>5500 m): a distinct risk stratum

At extreme altitude, physiology differs qualitatively from lower high-altitude tiers. Patients who develop PE during the early phase of extreme-altitude exposure show a markedly elevated risk of concurrent lower-extremity DVT (OR 16.3; 95% CI, 1.2 – 223.2) (Xiong et al., 2024) and shorter thrombus dissolution times (Wu et al., 2025). The synergy with HAPE creates a ‘perfect storm’ of severe hypoxia, prolonged immobilization, and excessive inflammation (Liu et al., 2023). Clinicians should regard extreme altitude as a separate risk stratum requiring aggressive prophylaxis.

## 3 Pathophysiological mechanisms: an integrated multi-stressor framework

**Figure 1** summarizes the HIF-centered multi-stressor mechanistic model. High altitude is an established independent VTE risk factor. We now examine the underlying pathophysiological mechanisms, in which HIF pathway activation serves as a plausible initiating hub of this complex regulatory network (Tourn et al., 2025). Epidemiological and mechanistic evidence converge to support a robust association: after adjusting for confounding factors, the association between high altitude and VTE remains statistically significant (Broggi et al., 2021; Yang et al., 2022; Plancher et al., 2025); gene expression studies have shown upregulation of ten genes including CDH18, FGA, and EDNBR in patients with high-altitude VTE (Srivastava et al., 2020); and epigenetic evidence indicates that hypoxia-mediated DNA methylation modifications can affect the expression of susceptibility genes (Arya et al., 2026).

### 3.1 Central regulatory role of the HIF pathway

The HIF pathway serves as the central mediator connecting high-altitude hypoxia to systemic prothrombotic responses, and is the core regulatory mechanism underlying multi-system hemostatic imbalance (Tourn et al., 2025). Under hypoxic conditions, the degradation of HIF-1α and HIF-2α is inhibited; HIF-2α is the key isoform mediating erythropoiesis and vascular adaptation to high altitudes, and the reduced degradation leads to nuclear accumulation of both isoforms and their heterodimerization with HIF-1β, thereby initiating transcription of downstream target genes (Li et al., 2022). Low temperature, a key environmental factor that synergizes with hypoxia in high-altitude regions, enhances the stability of HIF-1α (Li et al., 2022) and drives the switch of endothelial cell phenotype to a prothrombotic state. Activation of the HIF pathway upregulates the expression of erythropoietin (EPO), which induces secondary erythrocytosis, and simultaneously modulates the expression of tissue factor (TF), plasminogen activator inhibitor-1 (PAI-1), and other coagulation-related genes, thus promoting a prothrombotic state through multiple molecular pathways (Tourn et al., 2025).

### 3.2 Imbalance of coagulation-anticoagulation-fibrinolysis systems

High-altitude hypoxia induces a prothrombotic shift in systemic hemostatic balance. In the coagulation cascade, healthy volunteers have a substantial rise in plasma factor VIII and vWF after high-altitude exercise, as well as an increase in peak thrombin level and endogenous thrombin potential (ETP) (Ninivaggi et al., 2024). Equally, the plasma fibrinogen and fibrin degradation product are higher in TKA patients at high altitude, a sign that the coagulation system is activated with secondary hyperfibrinolysis (Zhao et al., 2023). In high-altitude hypoxia, the anticoagulant system is disturbed: with hypoxia, the expression of protein S, a central anticoagulant protein, is also lowered (Majumder and Nguyen, 2021); moreover, the activated partial thromboplastin time (APTT) of patients with severe HAPE who develop VTE is significantly reduced (Liu et al., 2023). Fibrinolysis is inhibited via HIF-upregulated PAI-1 expression (Ninivaggi et al., 2024). Collectively, increased procoagulant factors, reduced anticoagulant activity, and impaired fibrinolysis constitute the hematological basis of the high-altitude prothrombotic phenotype (Ninivaggi et al., 2024).

### 3.3 Endothelial dysfunction

The target cells that mostly ensure vascular coagulation-anticoagulation homeostasis are the vascular endothelial cells. Specifically, hypoxia at high altitudes disrupts endothelial anticoagulant-prothrombotic balance by causing direct cellular damage and inflammation, and resulting in a prothrombotic phenotypic shift in endothelial cells (Fan et al., 2020). Indeed, healthy volunteers show elevated plasma vWF activity after a short exposure to high altitude (Jacobson et al., 2024). Chronic hypoxia and oxidative stress uncouple endothelial nitric oxide synthase (eNOS), which reduces nitric oxide (NO) bioavailability and at the same time boosts the release of endothelin-1 (ET-1), a powerful constrictor of the vessel (Tremblay et al., 2020). Notably, hypoxia increases the expression of TF on the endothelial cell surface, which quickly activates the extrinsic coagulation cascade when vascular injury occurs (Yu et al., 2020). This endothelial damage as a result of hypoxia is a central element of the Virchow triad in pathogenesis of venous thrombosis (Schulman et al., 2024).

### 3.4 Hemorheological abnormalities and hypercoagulability

Secondary erythrocytosis—defined at high altitudes by the diagnostic criteria of hemoglobin >19 g/dL in men and >17 g/dL in women—is the primary cause of hemorheological abnormalities in high-altitude environments. HIF-EPO axis stimulation leads to erythropoiesis, and this effect is found in patients with high-altitude CVST (Yang et al., 2023) as well as extreme-altitude PE (Wu et al., 2025). Secondary erythrocytosis has a direct effect on whole blood viscosity, slowing venous blood flow and worsening venous stasis—in keeping with the stasis component of the Virchow triad of venous thrombosis (Tourn et al., 2025). Moreover, the elevated erythrocyte quantity could have procoagulant properties because of externalization of phosphatidylserine in the erythrocyte membrane (Tourn et al., 2025).

### 3.5 Amplification by inflammation and oxidative stress

Inflammation and oxidative stress are the primary amplifiers of thrombotic events at high altitude (Li et al., 2022): hypoxia-induced reactive oxygen species (ROS) directly damage endothelial cell membranes (Li et al., 2022) and trigger activation of the nuclear factor kappa B (NF-κB) pathway, which results in IL-6 release, TNF-α release, and other proinflammatory cytokines (Majumder and Nguyen, 2021). These cytokines activate TF of endothelial cells, and at the same time suppress the role of protein S as an anticoagulant (Majumder and Nguyen, 2021), which constitutes a positive feedback loop between inflammation and coagulation. This feedback loop is particularly evident in high-altitude VTE. Thrombosis stimulates inflammatory cells, which release proinflammatory mediators that in turn amplify coagulation signals, creating a vicious cycle of thrombosis-inflammation that accelerates pathogenesis in severe cases (Nicolai et al., 2020). Thrombotic events of acute PE in animal models are associated with an inflammatory response of leukocyte infiltration and strong secretion of selectins and proinflammatory cytokines (Imiela et al., 2024). Inflammatory conditions also stimulate endothelial cells and platelets, and stimulate neutrophil extracellular traps (NETs) development; NETs form a procoagulant scaffold recruiting erythrocytes and platelets, enhancing the coagulation cascade (Schulman et al., 2024).

### 3.6 Hemodynamic alterations and venous stasis

Hypoxic pulmonary hypertension (HPH) and peripheral venous stasis have a synergistic effect that greatly endangers the development of VTE in high-altitude settings (Broggi et al., 2021). Chronic hypoxia causes pulmonary vasoconstriction, vascular remodeling, right ventricular afterload (then right ventricular dysfunction), and elevated pulmonary arterial pressure impairs systemic venous return, raising central venous pressure (CVP), and exacerbates peripheral venous stasis (Liu et al., 2023). Approximately one in four patients with severe HAPE has both pulmonary hypertension and right heart dysfunction, which is one of the major factors contributing to the dramatically high VTE rate among this group of patients (Liu et al., 2023). A microenvironment hemodynamically favoring thrombotic formation occurs in the case of elevated CVP and systemic venous congestion with lowered local venous blood flow due to dysfunction of the right heart.

### 3.7 Integrated multi-stressor model

Beyond hypoxia, low temperature and dehydration dominate the high-altitude environment. Dehydration is common at high altitude (Jacobson et al., 2024). Low humidity, increased insensible sweat loss, and hypoxia-blunted thirst sensation collectively reduce fluid intake and promote hemoconcentration (Jacobson et al., 2024). Air travel studies have revealed a high positive relationship between urine osmolality, which is a validated biomarker of dehydration, and plasma vWF, implying that dehydration can cause the increase of endothelial activation and procoagulant tendency (Jacobson et al., 2024). Peripheral vasoconstriction caused by low temperature will decrease blood flow in the limbs and worsen the state of venous stasis (Li et al., 2022).

### 3.8 Genetic susceptibility and epigenetic regulation

The fundamental molecular components of the high level of disparity in VTE vulnerability between populations residing in high-altitude conditions are interindividual genetic and epigenetic differences. This heterogeneity is the main factor to explain why traditional VTE risk assessment tools have limited predictive values in high-altitude environments (Yang et al., 2022). Among known susceptibility genes, angiogenin (ANG) gene polymorphisms may exacerbate endothelial dysfunction by altering angiogenesis, while angiotensin-converting enzyme (ACE) gene variants may amplify hypoxia-induced procoagulant responses through modulation of the renin-angiotensin system (Yang et al., 2022). The long non-coding RNAs LINC00659 and UXT-AS1, as well as platelet glycoprotein IV (GP4), may be associated with high-altitude VTE, but their specific regulatory mechanisms remain to be elucidated (Yang et al., 2022). Gene expression profiling studies have identified ten genes (including CDH18, FGA, EDNBR, GATA2, and MAPK9) that are significantly upregulated in patients with high-altitude VTE compared with those with low-altitude VTE (Srivastava et al., 2020). Epigenetically, hypoxia may alter the DNA methylation status of CDH18, FGA, and other coagulation-related genes (Arya et al., 2026), leading to their chronic upregulation. Overexpression of CDH18 significantly enhances the adhesive capacity of vascular endothelial cells, while upregulation of FGA directly promotes hepatic fibrinogen synthesis; these changes collectively form the molecular basis for individual susceptibility to high-altitude-specific thrombotic events (Srivastava et al., 2020; Arya et al., 2026). The interaction between inherited thrombophilia, such as Factor V Leiden, prothrombin G20210A mutation, or antiphospholipid antibodies—and high-altitude hypoxia remains unexplored. These conditions may amplify hypoxia-induced prothrombotic stress, but no high-altitude cohort has quantified this interaction.

## 4 Development of high-altitude-specific risk assessment systems

### 4.1 Limitations of conventional risk scores at high altitude

The traditional Caprini and Padua scores, which are prevalently used to assess the risk of VTE at sea level, are significantly restricted in high-altitude locations. These instruments do not include altitude and its physiological perturbations (hypoxia, hemoconcentration) as one of the fundamental risk variables, which can result in underestimating baseline VTE risk in high-altitude populations (Jones et al., 2022; Shi et al., 2025). TKA studies have confirmed that an altitude above 1219 m is an independent risk factor for VTE at 30 and 90 days post-surgery, separate from traditional surgical and patient-related risk factors (Plancher et al., 2025). This highlights the need for surgeons to integrate altitude into preoperative risk assessment and tailor thromboprophylaxis protocols accordingly. The Caprini score, with its comprehensive inclusion of clinical variables, retains some predictive value in hospitalized patients at high altitudes but requires modification by incorporating altitude-specific physiological parameters to improve its predictive efficacy (Shi et al., 2025).

### 4.2 Core components of the proposed three-dimensional model

#### 4.2.1 Altitude and physiological compensation

Developing a high-altitude-specific VTE risk assessment system requires the quantification and integration of altitude-specific indicators. Altitude itself is the most direct indicator: patients with PE at extreme high altitudes (a mean of 5041 m) display distinct clinical characteristics compared with low-altitude counterparts, including shorter thrombus dissolution times (Wu et al., 2025); patients who develop PE during the early phase of extreme altitude exposure also have an elevated risk of concurrent lower-extremity DVT (Xiong et al., 2024). Hypoxia-induced physiological compensatory responses are critical mediators linking altitude exposure and VTE development: elevated HCT and hemoglobin levels are well-recognized prothrombotic factors (Yang et al., 2023; Wu et al., 2025); pulse oxygen saturation (SpO_2_) directly reflects systemic oxygenation status and correlates with hypoxia-induced endothelial injury and coagulation activation (Liu et al., 2023). Effective risk assessment models must integrate altitude classification, SpO_2_, HCT/hemoglobin levels, and a history of high-altitude illness with appropriate weighting to precisely identify high-risk individuals. Altitude classification and SpO_2_ should be assigned higher weighting, as they directly reflect the severity of hypoxic exposure and are the core drivers of high-altitude VTE.

#### 4.2.2 Biomarker adaptation: validated and exploratory markers

D-dimer is a commonly used serum biomarker for VTE screening, but hypoxic conditions at high altitudes can cause a physiological elevation in its baseline levels (Liu et al., 2023; Jacobson et al., 2024). This necessitates the establishment of altitude-specific diagnostic cutoffs for D-dimer to avoid misinterpretation (validated clinical marker). Combining D-dimer testing with the Caprini score has been shown to improve predictive performance for high-altitude VTE, and this approach has been validated in high-altitude populations (Shi et al., 2025).

Exploration of novel biomarkers is essential for a deeper understanding of high-altitude VTE mechanisms and risk stratification. NETs are activated in hypoxic environments and promote thrombosis through two core mechanisms: forming a procoagulant scaffold and releasing TF to activate the extrinsic coagulation pathway (Tourn et al., 2025); DNA methylation may participate in the modulation of VTE susceptibility induced by high-altitude hypoxia (Arya et al., 2026); microRNA (miRNA) expression profiles correlate with genetic susceptibility to VTE (Yang et al., 2022); metabolomic studies have identified elevated uric acid levels in patients with PE at extreme high altitudes as a potential exploratory risk marker, though this finding requires validation in large cohort studies (Wu et al., 2025) (exploratory candidates requiring prospective validation). The specificity, sensitivity as well as incremental predictive value of these novel biomarkers in high-altitude VTE risk assessment models need to be verified in future studies. Pending prospective validation, we propose preliminary altitude-adjusted D-dimer cutoffs: 0.8 mg/L at 2500–3500 m and 0.6 mg/L at 3500–5500 m. These values should be integrated with clinical probability assessments rather than used as standalone thresholds.

### 4.3 Model development, validation, and clinical integration

To be able to prevent and manage high-altitude VTE with precision, it is important to integrate conventional VTE risk factors, high-altitude core indicators, and modified biomarkers in multidimensional models. The combined Caprini-D-dimer model has demonstrated superior predictive performance in hospitalized patients at high altitudes, with an area under the curve (AUC) of 0.89; this improves to an AUC of 0.904 when excluding patients receiving pharmacological thromboprophylaxis, and the model has also undergone robust external validation with an AUC of 0.86 (Shi et al., 2025). Future risk assessment models should further incorporate altitude classification, SpO_2_, hematological parameters, and a history of high-altitude illness. Machine learning algorithms (e.g., random forests, gradient boosting) offer distinct advantages over conventional logistic regression, as they can automatically identify complex interactions between variables to optimize predictive performance (Jones et al., 2022; Yang et al., 2024). Integration of these models into clinical decision support systems could generate real-time, individualized risk stratification and prevention recommendations for high-risk clinical subgroups—such as orthopedic surgical patients, trauma patients, and AECOPD patients with rapid ascent to high altitudes (Jones et al., 2022; Yang et al., 2024). This requires prospective, multi-center, large-scale cohort studies for model training, internal validation, and iterative optimization; after model construction, multi-center, cross-altitude external validation is a prerequisite for its clinical application.

**Figure 2:**
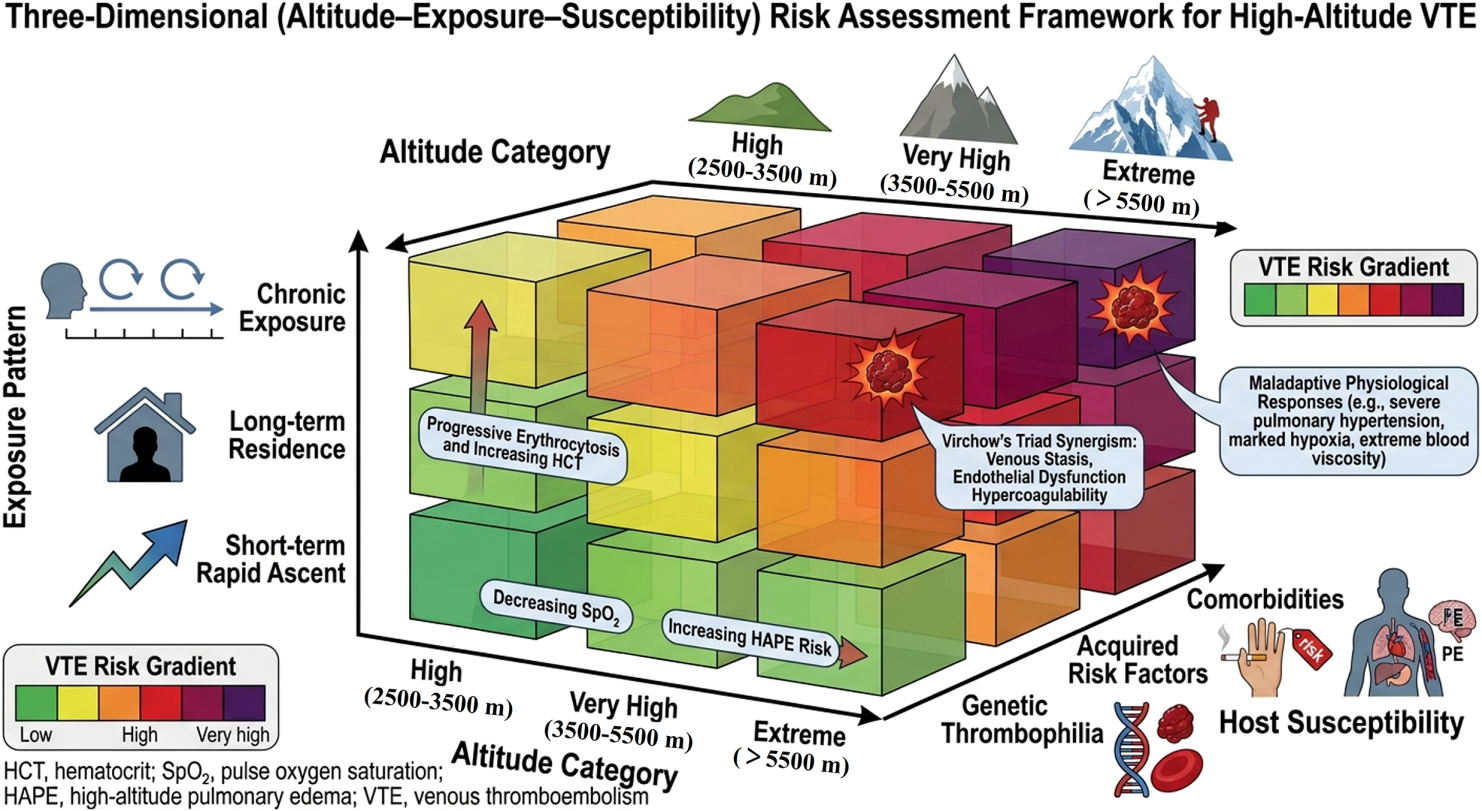
Three-dimensional (altitude–exposure–susceptibility) risk assessment framework for high-altitude VTE. The x-axis represents altitude category (high: 2500–3500 m; very high: 3500–5500 m; extreme: >5500 m), the y-axis exposure pattern (short-term rapid ascent, long-term residence, chronic exposure), and the z-axis host susceptibility (genetic thrombophilia, acquired risk factors, comorbidities). Risk intensity increases from green (low) to purple (very high). Annotated physiological drivers include decreasing pulse oxygen saturation (SpO_2_) and increasing high-altitude pulmonary edema (HAPE) risk with rapid ascent, progressive erythrocytosis with long-term residence, and Virchow triad synergism at high-risk intersections. SpO_2_, pulse oxygen saturation; HAPE, high-altitude pulmonary edema.

The proposed model assigns preliminary weights derived from existing evidence: altitude category (40%, based on documented incidence gradients), SpO_2_ (30%, reflecting hypoxia severity), history of high-altitude illness (20%), and hematological parameters including HCT/hemoglobin (10%). Variable selection should prioritize physiologically plausible markers with demonstrated univariate associations. Model training requires prospective multicenter cohorts across altitudes (2500–5500 m), with internal validation (bootstrapping) and external validation across geographic regions. Performance metrics include discrimination (AUC-ROC), calibration (Hosmer-Lemeshow test), and decision curve analysis. Upon validation, integration into electronic health records could enable real-time risk stratification. **Figure 2** illustrates the proposed three-dimensional risk framework.

## 5 Comprehensive prevention and management strategies

High-altitude-specific risk assessment models provide a tool for precise identification of high-risk populations, which supports the development of a stratified, efficient prevention and management system covering prophylaxis, diagnosis, and treatment of high-altitude VTE.

### 5.1 Primary prevention: risk-stratified comprehensive approaches

Non-pharmacological interventions form the cornerstone of primary prophylaxis for high-altitude VTE. Adequate hydration is critical: the elevated risk of dehydration at high altitudes can directly increase blood viscosity and indirectly activate the coagulation system (Yang et al., 2023; Jacobson et al., 2024). Healthy volunteers exhibit elevated urine osmolality following one hour of simulated high-altitude flight, with a positive correlation between urine osmolality and plasma vWF activity (Jacobson et al., 2024). Regular limb activity and physical thromboprophylaxis are equally important: hospitalized patients with severe HAPE have a VTE incidence of 38.9%, in part due to prolonged immobilization (Liu et al., 2023); for patients unable to mobilize independently, intermittent pneumatic compression devices or graduated compression stockings should be considered. Gradual altitude acclimatization is another key non-pharmacological measure: the standard international stepwise ascent protocol (≤300–500 m/day above 2500 m, with a rest day every 3–4 days) effectively prevents acute mountain sickness and associated thrombotic events (Liu et al., 2023). Notably, all patients with severe HAPE report a rapid first-time ascent to high altitudes, and rapid ascent has been associated with the development of multi-site thrombotic events within days (Gan et al., 2025).

Pharmacological prevention: LMWH is the first-line prophylactic agent for high-altitude VTE (Qiong et al., 2025). A prospective study in ICUs in Tibet demonstrated that standardized LMWH prophylaxis reduced the incidence of VTE from 42.9% to 35.9%, with no significant increase in bleeding events (Qiong et al., 2025). This is consistent with evidence of the benefit of anticoagulation prophylaxis in postoperative patients at high altitudes (Plancher et al., 2025). TKA clinical protocols must integrate altitude factors to develop individualized thromboprophylaxis strategies (Plancher et al., 2025). Direct oral anticoagulants (DOACs) have limited clinical data in high-altitude populations but may be suitable for specific clinical situations due to their convenient administration and no need for regular laboratory monitoring; however, their pharmacokinetic characteristics in high-altitude populations remain unclear, requiring individualized assessment before use. Antiplatelet agents (e.g., aspirin) remain controversial for high-altitude VTE prophylaxis: some studies suggest their potential as adjunctive thromboprophylaxis in athletic populations (Diep and Garcia, 2020), but most clinical evidence indicates that the core mechanisms of high-altitude VTE involve thrombin activation rather than platelet aggregation alone (Majumder and Nguyen, 2021). This makes antiplatelet agents less effective in comparison to LMWH or DOACs in the high-altitude VTE prophylaxis (Majumder and Nguyen, 2021). The use of these agents is therefore only suggested as a second option when anticoagulation cannot be used because of a high propensity to bleeding. Enhanced prevention of high-risk groups: Patients with a history of VTE, hereditary thrombophilia (e.g. factor V Leiden mutation) or post-COVID-19 VTE/myocarditis are extremely high-risk populations in high-altitude settings (Luks and Grissom, 2021). For these individuals, prophylaxis should start early and prophylaxis period may require some extension than the conventional interventions.

### 5.2 Diagnostic strategies: optimized approaches for high-altitude settings

**Figure 3:**
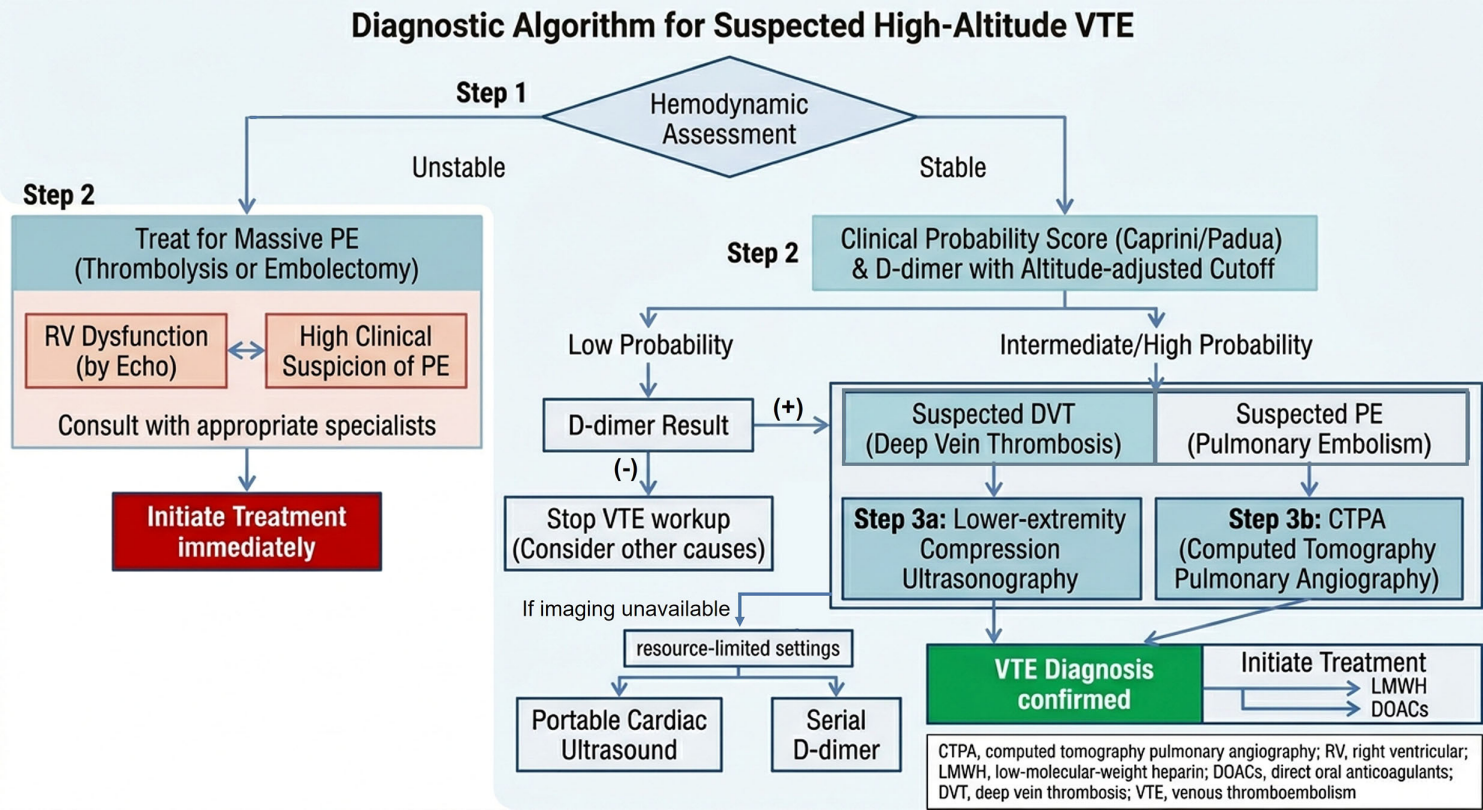
Diagnostic algorithm for suspected high-altitude VTE. Step 1 assesses hemodynamic stability. Unstable patients with suspected massive pulmonary embolism (PE) receive immediate thrombolysis or embolectomy guided by echocardiographic right ventricular (RV) dysfunction. Step 2 (stable patients): apply a clinical probability score (Caprini or Padua) with D-dimer using altitude-adjusted cutoffs; low-probability patients with negative D-dimer may discontinue workup. Intermediate- or high-probability patients proceed to Step 3 imaging—lower-extremity compression ultrasonography for suspected deep vein thrombosis (DVT) (3a), or computed tomography pulmonary angiography (CTPA) for suspected PE (3b). In resource-limited settings, portable cardiac ultrasound and serial D-dimer are alternatives. Confirmed VTE prompts therapeutic anticoagulation with low-molecular-weight heparin (LMWH) or direct oral anticoagulants (DOACs). CTPA, computed tomography pulmonary angiography; DVT, deep vein thrombosis; LMWH, low-molecular-weight heparin; DOACs, direct oral anticoagulants.

**Figure 3** presents a stepwise diagnostic algorithm for resource-limited high-altitude settings. Diagnosis of high-altitude VTE entails a solution to the “symptom overlap trap” between VTE and altitude-specific disorders. Clinicians are advised to have a high index of suspicion of VTE among patients with chronic or unexplainable dyspnea, chest pains or any other associated symptoms following high-altitude exposure. Interpretation of D-dimer results requires caution: high-altitude exposure itself can cause a physiological elevation in D-dimer levels, so results must be integrated with clinical probability assessments such as the Caprini score (Shi et al., 2025). Imaging studies are the gold standard for confirming a VTE diagnosis. Lower-extremity compression ultrasonography is the preferred method for diagnosing DVT, while computed tomography pulmonary angiography (CTPA) is the gold standard for diagnosing PE (Liu et al., 2023). However, in high-altitude settings with limited medical resources where CTPA is unavailable, portable bedside cardiac ultrasound can be used to assess right ventricular function as an auxiliary tool for severity evaluation (Liu et al., 2023). Among AECOPD patients at high altitudes, a Padua score >3, concurrent DVT, and D-dimer >1 mg/L are independent risk factors for PE (Yang et al., 2024). This indicates the need for routine VTE screening in this population, including Padua score assessment, D-dimer testing, and lower extremity venous ultrasonography. In resource-limited high-altitude settings, a pragmatic triage strategy combining clinical symptoms, D-dimer testing, and portable bedside ultrasound may be employed (Liu et al., 2023; Yang et al., 2024).

### 5.3 Acute-phase treatment: high-altitude-specific adjustments

The core of acute-phase treatment for high-altitude VTE is balancing antithrombotic efficacy and bleeding risk. High-altitude-induced hypercoagulable states mandate the prompt initiation of adequate anticoagulation—this is the fundamental principle of acute-phase treatment, with close attention to the effects of high altitude on drug metabolism and the complexity of coagulation function (Zhao et al., 2023). TKA studies have shown altered coagulation and fibrinolysis parameters in high-altitude patients, which may increase the risk of bleeding (Zhao et al., 2023). The initiation of therapeutic-dose anticoagulation or thrombolysis therefore requires a careful bleeding risk assessment, with particular attention to HCT levels and hydration status. Thrombolysis and interventional therapy for high-risk PE require a rigorous risk-benefit assessment. Patients at high altitudes frequently exhibit hemoconcentration and secondary erythrocytosis, which may increase the risk of intracranial hemorrhage (ICH) (Wu et al., 2025). Some studies have identified shorter thrombus dissolution times in patients with PE at extreme high altitudes compared with low-altitude counterparts (Wu et al., 2025), which may reflect the younger age and higher fibrinolytic activity of this population, or a hypoxia-induced “compensatory activation” of the fibrinolytic system. However, this does not mean that treatment courses can be simplified or shortened; individualized adjustment of anticoagulation duration based on bleeding risk assessment remains essential to avoid premature discontinuation and subsequent thrombosis recurrence. In the absence of high-altitude-specific anticoagulant dosing guidelines, clinical decisions should be guided by serial monitoring of coagulation parameters (APTT, INR, anti-Xa activity) and individualized risk-benefit assessment. The pharmacokinetics of DOAC reversal agents, including andexanet alfa for factor Xa inhibitors and idarucizumab for dabigatran—have not been studied in hypobaric hypoxia. Hemoconcentration and altered hepatic perfusion at high altitude may affect their distribution and efficacy, representing a critical safety gap.

### 5.4 Long-term management and complication prevention

Long-term management of high-altitude VTE focuses on individualized decisions regarding the duration of anticoagulation. Standard anticoagulation courses for VTE typically range from 3 to 6 months; however, whether high altitude—as a persistent prothrombotic risk factor—warrants extended anticoagulation courses remains undefined. This decision requires a comprehensive evaluation based on recurrence risk, bleeding risk, and patient preferences, with regular monitoring for bleeding manifestations and surveillance of laboratory coagulation parameters. Chronic thromboembolic pulmonary hypertension (CTEPH) is a severe, potentially fatal long-term complication of PE. High-altitude hypoxia itself can elevate pulmonary arterial pressure, which may mask or aggravate CTEPH (Liu et al., 2023). Clinicians should maintain a high vigilance for CTEPH in patients with persistent dyspnea or reduced exercise tolerance following an acute PE. Systematic CTEPH screening is recommended 3–6 months after the completion of anticoagulation, including echocardiography, ventilation/perfusion scanning, or right heart catheterization (Liu et al., 2023). Secondary erythrocytosis, a common finding in long-term high-altitude residents, may exacerbate the risk of thrombosis recurrence by increasing blood viscosity and aggravating the procoagulant state (Yang et al., 2023). For these individuals, long-term management may include phlebotomy or pharmacotherapy to maintain a hematocrit (HCT) <55% (Yang et al., 2023).

### 5.5 Altitude management and descent decisions

Altitude management is an important adjunct to the acute-phase treatment of high-altitude VTE, with significant individualized needs across different exposure populations. Timely descent to lower altitudes is a critical adjuvant therapeutic measure for severe high-altitude VTE, with the core objectives of relieving hypoxemia, reducing pulmonary arterial pressure, and improving systemic oxygenation (Nimmatoori et al., 2020). Indications for urgent descent include massive PE, severe hypoxemia (SpO_2_<75% with no improvement after supplemental oxygen), and comorbid HAPE (Liu et al., 2023). For lowland migrants who develop acute VTE after rapid ascent to extreme altitudes, descent to lower elevations should be prioritized (Nimmatoori et al., 2020); for high-altitude natives with chronic VTE and hemodynamic stability, *in-situ* treatment with intensified oxygen support is feasible, with careful monitoring of HCT levels (Shi et al., 2025). The fundamental principles of early and safe transport should be followed: hemodynamically unstable patients with severe PE should be urgently transported under medical control after medical hemodynamic stabilization, and in stable patients, risks of the transport should be considered and balanced against the risk of living at high altitudes. The descent planning should be thorough and medical escort with anticoagulation needs to be given at times of necessity (Gan et al., 2025). In situations in which immediate descent is no longer possible, the priority should be given to intensified oxygen support (maintaining SpO_2_≥90%) and the use of convenient anticoagulant drugs such as DOACs; close attention to both the state of hemodynamics and coagulation parameters should also be paid.

Descent logistics require operational and medicolegal planning. Hemodynamically unstable patients with massive PE require helicopter evacuation when terrain and weather permit; ground transport over mountainous roads risks further hypoxemia. Clinicians must document the rationale for descent timing and transport mode, as delayed decisions may carry liability. When evacuation is impossible, *in-situ* stabilization with supplemental oxygen and anticoagulation remains standard, with detailed documentation of resource constraints. **Table 2** summarizes key recommendations for VTE prevention, diagnosis, and management stratified by altitude and exposure type.

**Table 2:** Key recommendations for VTE prevention, diagnosis, and management at high altitude.

Altitude category	Exposure type	Prevention	Diagnosis	Treatment
2500–3500 m	Short-term	Hydration, graded ascent; LMWH for high-risk surgical patients	D-dimer plus clinical score; compression ultrasonography	Standard anticoagulation
3500–5500 m	Rapid ascent	Aggressive hydration, rest days; consider LMWH prophylaxis	Altitude-adjusted D-dimer; portable ultrasound if CTPA unavailable	Therapeutic anticoagulation or thrombolysis/embolectomy for massive PE; monitor bleeding risk
>5500 m	Any (differentiated by population)	Mandatory LMWH for high-risk groups; strict acclimatization (avoid rapid ascent)	D-dimer plus bedside ultrasound; urgent CTPA if available	Urgent descent for massive PE; individualized anticoagulation duration

Recommendations are based on limited evidence and expert consensus; clinical judgment should always guide individual patient management.

## 6 Individualized management of special populations

### 6.1 High-altitude athletes and outdoor expedition populations

The risk of VTE among high-altitude athletes and participants of outdoor expeditions is also unique: moderate training alters physical endurance through the production of more erythrocytes, whereas overtraining can also lead to dehydration and extended immobility increasing the risk of thrombosis (Wisniewska et al., 2020; Cerda-Kohler et al., 2022). Moderate-altitude training and its impact on the maximum exercise performance are not clear either (Wisniewska et al., 2020). The risk of PE is greatly increased at very high altitudes (>5000 m), especially during the initial period of exposure, and patients with PE at such high altitudes are more likely to have other lower-extremity DVT (Xiong et al., 2024). The advantages of prophylaxis (e.g., LMWH) of this group should be weighed with the dose of bleeding, but the existing clinical data are scarce (Xiong et al., 2024). These populations should increase self-health monitoring and specifically focus on common symptoms of VTE, including swelling and pain in one of the limbs, dyspnea that has no apparent causes, and chest pain. The development of HAPE in these individuals should raise a high suspicion for concomitant VTE, with prompt completion of relevant diagnostic tests (Liu et al., 2023). Before embarking on extended high-altitude expeditions, a baseline VTE risk assessment should be completed—including coagulation function testing and, if available, thrombotic susceptibility gene testing—and an individualized prevention plan developed (e.g., strict hydration protocols, intermittent limb activity, short-term anticoagulation when necessary).

### 6.2 Patients with comorbid conditions

High-altitude hypoxia amplifies the hypercoagulable tendencies associated with underlying chronic diseases. Patients with chronic kidney disease (CKD) have a substantially elevated risk of VTE at high altitudes due to poor hypoxia tolerance and disordered water-electrolyte metabolism (Furuto et al., 2020); oncology patients, already in a hypercoagulable state due to malignancy and chemotherapy, require careful assessment of the additive prothrombotic effects of high-altitude-related stressors (Yang et al., 2022). Post-COVID-19 patients with a history of VTE or myocarditis require a comprehensive medical evaluation of cardiopulmonary function and coagulation function before planning any high-altitude travel; the trip may need to be adjusted or postponed if cardiopulmonary or coagulation abnormalities are identified (Luks and Grissom, 2021). Particular attention should be paid to drug interactions: patients with chronic conditions frequently require long-term antiplatelet or anticoagulant medications, and high altitude may potentially affect drug metabolism or efficacy. When adding prophylactic anticoagulation for high-altitude VTE, careful assessment of drug-drug interactions and bleeding risk is essential; a multidisciplinary collaborative approach is recommended to develop individualized medication plans. Patients with known thrombophilia require hematology consultation before high-altitude exposure.

### 6.3 Perioperative and critically ill patients

High altitude is an independent risk factor for VTE following major orthopedic surgery, and it exerts a synergistic effect with perioperative anesthesia, surgical trauma, and postoperative prolonged immobilization to significantly elevate VTE risk (Broggi et al., 2021; Jones et al., 2022; Plancher et al., 2025). Following TKA, patients at altitudes above 1219 m have increased 30- and 90-day rates of VTE and DVT (Plancher et al., 2025); postoperative VTE risks are similarly increased in patients undergoing tibial plateau fracture surgery (Jones et al., 2022) and pelvic and acetabular fracture surgery (Broggi et al., 2021). The underlying mechanisms involve the synergy of surgical trauma, anesthesia, immobilization, and high-altitude-induced hypercoagulability (Tourn et al., 2025). For patients undergoing major orthopedic surgery (TKA, tibial plateau fracture, pelvic fracture) in high-altitude regions, a high-altitude-specific VTE risk assessment should be completed preoperatively; combined mechanical and pharmacological thromboprophylaxis should be initiated as early as possible postoperatively, with the duration of pharmacological prophylaxis appropriately extended (Plancher et al., 2025). Critically ill patient management: A prospective study in ICUs in Tibet demonstrated that standardized LMWH prophylaxis effectively reduced VTE incidence without increasing bleeding risk (Qiong et al., 2025). More risk-stratified VTE prevention measures are thus necessary in high-altitude critically ill patients, such as a combination of mechanical prevention of VTE with LMWH pharmacological prevention, as well as the serial assessment of coagulation function and bleeding (Qiong et al., 2025).

### 6.4 Pregnant women, elderly, and pediatric populations

Pregnant women: Management requires a careful balance of maternal and fetal safety. LMWH prophylaxis can be used in the second trimester after joint evaluation by obstetric and thrombotic specialists (Wu et al., 2022); dose reduction based on body weight is required in the third trimester, with close monitoring for bleeding tendencies (e.g., vaginal bleeding, gingival bleeding) (Wu et al., 2022). High-altitude hypoxia may superimpose on the physiological hypercoagulability of pregnancy, significantly elevating VTE risk; however, large-sample research data on VTE prophylaxis and treatment in this population remain scarce, and clinical management should be based on individualized risk assessment (Wu et al., 2022). Elderly populations: Elderly patients frequently have multiple chronic comorbidities and declining physical function; reduced activity and an elevated risk of dehydration in high-altitude environments collectively increase their VTE risk (Quan et al., 2023). The core focus of VTE prevention for elderly high-altitude patients includes maintaining adequate hydration, avoiding prolonged bed rest, and developing individualized anticoagulation plans that take into account the impact of impaired liver and kidney function on drug metabolism (Quan et al., 2023).

Pediatric populations: Pediatric VTE incidence is low overall, but high-altitude exposure can trigger severe thrombotic events including CVST (Gan et al., 2025). No pediatric registry data from high-altitude regions have been published; existing evidence is limited to case reports and small series. Key management principles include strict avoidance of adult anticoagulant doses for pediatric patients; LMWH doses must be accurately calculated based on body weight (Gan et al., 2025). DOACs are not recommended for routine use in pediatric high-altitude populations due to insufficient safety data, and can only be used cautiously after evaluation by pediatric thrombotic specialists in special clinical situations.

### 6.5 High-altitude natives vs. lowland migrants

High-altitude natives and lowland migrants have inherently different core VTE risks, requiring differentiated management strategies. High-altitude natives have undergone generational adaptive changes to hypoxic environments, with elevated baseline hemoglobin and HCT levels as physiological compensatory mechanisms (Shi et al., 2025). However, chronic erythrocytosis may increase blood viscosity and elevate thrombosis risk (Yang et al., 2023), so regular HCT monitoring is required; if HCT >55%, phlebotomy or pharmacological intervention can be used to reduce blood viscosity and lower thrombosis risk (Yang et al., 2023). In contrast, lowland migrants—particularly those who ascend rapidly—experience acute high-altitude maladaptation, characterized by excessive sympathetic activation, dehydration, and enhanced inflammatory responses (Jacobson et al., 2024). These are the core reasons for their substantially elevated early VTE risk after high-altitude exposure (Jacobson et al., 2024). Case reports have documented multi-site thrombotic events within days of rapid ascent to high altitudes, highlighting the acute VTE risk during the acclimatization period (Gan et al., 2025). For lowland migrants, targeted risk mitigation is critical during the early acclimatization phase, including enhanced health education, strict hydration protocols (≥3000 mL of daily water intake), and early symptom monitoring (Shi et al., 2025). For migrants who ascend rapidly to high altitudes, intensified hydration, avoidance of strenuous activity, and intermittent limb activity are recommended during the 7–14 day acclimatization period; short-term prophylactic LMWH may be considered for high-risk migrants after individual risk-benefit assessment (Shi et al., 2025). High-altitude-specific parameters in risk prediction models may additionally help predict high-risk persons in both groups, thus the prevention of high-altitude VTE may be done precisely (Shi et al., 2025).

## 7 Research limitations and future directions

### 7.1 Current evidence gaps

Although much has been learnt about high-altitude VTE, observable gaps persist at the epidemiological, mechanistic, and clinical levels. Epidemiologically, a lack of large-scale prospective cohort studies with geographically diverse high-altitude areas, variable amounts of exposure, and different populations (i.e., tourists vs. long-term residents) exists (Yang et al., 2022). This makes it difficult to clarify the true incidence and complete risk spectrum of high-altitude VTE. Mechanistically, the key molecular targets within the hypoxia-coagulation-inflammation-oxidative stress regulatory network remain undefined (Tourn et al., 2025), which hinders the development of targeted intervention drugs; the synergistic effects of genetic and epigenetic mechanisms on high-altitude VTE susceptibility also remain to be further elucidated (Arya et al., 2026). Clinically, safety and efficacy data for anticoagulation in special high-altitude populations (e.g., pregnant women, pediatric patients) are insufficient (Wu et al., 2022; Gan et al., 2025); high-altitude-specific risk assessment models lack multi-center cross-regional validation (Shi et al., 2025); and standardized guidelines for anticoagulant dosing adjustments based on the pharmacokinetic characteristics of high-altitude populations are lacking (Plancher et al., 2025). This results in empirical clinical medication without robust evidence-based support.

Publication bias may inflate the perceived risk of high-altitude VTE, as positive associations are more likely to be published than null findings.

### 7.2 Translational research frontiers

To address these existing gaps, future translational research on high-altitude VTE should prioritize the following core directions: Conduct multi-center prospective cohort studies across different altitude regions to clarify the VTE risk spectrum in distinct populations and establish altitude-specific VTE incidence data (Yang et al., 2022); Use multi-omics technologies (genomics, epigenomics, metabolomics, proteomics) to screen for high-altitude VTE-specific molecular markers and key regulatory targets (Srivastava et al., 2020; Arya et al., 2026); Perform well-designed randomized controlled trials (RCTs) in special high-altitude populations to validate the safety and optimal dosing protocols of anticoagulants and novel thromboprophylactic agents (Qiong et al., 2025); Conduct multi-center validation and iterative optimization of high-altitude-specific VTE risk assessment models to improve their predictive accuracy and clinical applicability (Shi et al., 2025).

Multi-omics integration analysis will enable a systematic exploration of the molecular basis of high-altitude VTE: genomics for identifying functional loci of susceptibility genes such as ANG and ACE (Yang et al., 2022), epigenomics for analyzing hypoxia-mediated methylation regulation of coagulation-related genes (Arya et al., 2026), and metabolomics for validating the clinical value of potential markers such as uric acid (Wu et al., 2025). This will ultimately construct a multidimensional molecular network map of high-altitude VTE. The development of novel animal models that accurately simulate the dynamics of hypoxia-induced thrombosis in high-altitude environments is also critical (Tourn et al., 2025). From a therapeutic perspective, investigation of HIF pathway inhibitors (e.g., HIF-2α-specific inhibitors) for improving hypoxia-induced endothelial dysfunction and regulating the coagulation-inflammation balance is a promising direction—these inhibitors represent potential novel drugs for high-altitude VTE prevention and treatment, though their safety and efficacy in high-altitude populations remain unvalidated (Tourn et al., 2025). Additionally, the development of NET-targeted degrading agents or vWF activity-targeted interventions represents other promising targets for the precision prevention and treatment of high-altitude VTE (Jacobson et al., 2024; Schulman et al., 2024). Specifically, a priority RCT should compare prophylactic LMWH (enoxaparin 40 mg daily) versus a direct oral anticoagulant (e.g., apixaban 2.5 mg twice daily) in rapid ascenders (≤7 days) to 3500–5500 m, with a primary outcome of 30-day VTE incidence and secondary outcomes of major bleeding, altitude-specific D-dimer trajectories, and quality-adjusted survival.

### 7.3 Clinical practice advancement

The ultimate goal of high-altitude VTE research is to translate basic and translational research findings into clinical practice and public health policy. First, evidence-based high-altitude-specific VTE prevention and treatment guidelines or expert consensus are urgently needed (Shi et al., 2025). These guidelines should list altitude as an independent VTE risk factor and propose differentiated prevention and treatment recommendations based on altitude classification (high/very high/extreme) and exposure patterns (short-term/long-term/acute rapid ascent).

Second, the development and validation of risk prediction and diagnostic tools adapted to high-altitude scenarios are essential. The combined Caprini score and D-dimer model already demonstrates superior predictive value to single indicators in high-altitude populations (Shi et al., 2025); future studies should integrate novel biomarkers and clinical parameters using machine learning algorithms to develop more precise risk prediction models. Notably, given the limited medical resources in most high-altitude regions and the masking of VTE symptoms by altitude-related illnesses (Wu et al., 2022), the development of portable, rapid point-of-care diagnostic devices (e.g., portable D-dimer analyzers, mini ultrasound devices) suitable for primary care in high-altitude regions is critical (Liu et al., 2023). Handheld compression ultrasound systems (e.g., Butterfly iQ, Philips Lumify) and portable D-dimer analyzers (e.g., AQT90 FLEX) offer pragmatic alternatives in resource-limited settings, though high-altitude-specific validation is absent. A telemedicine diagnostic collaboration system should also be established to connect primary care institutions in high-altitude regions with specialized thrombosis centers, improving the diagnostic capacity of primary care for high-altitude VTE (Liu et al., 2023).

Lastly, public health prevention and control of high-altitude VTE should be strengthened (Qiong et al., 2025): Conduct systematic health education of high-altitude travelers, mountaineers, and migrant workers, focusing on the main preventive practices, such as hydration, gradual ascent, and early symptom detection of VTE (Liu et al., 2023; Jacobson et al., 2024); Implement the standard measures of VTE prevention in high-altitude health institutions, especially in the surgical and ICU departments (Qiong et al., 2025).

Key messages for high-altitude travelers.

- Hydration: Drink at least 3 liters of water daily; limit alcohol and caffeine.
- Ascent rate: Increase sleeping altitude by no more than 500 m per day above 2500 m; include a rest day every 3–4 days.
- Activity: Avoid prolonged immobility during travel; move legs regularly during long flights or drives.
- Warning signs: Seek immediate medical care for unexplained shortness of breath, chest pain, coughing blood, or one-sided leg swelling or pain.
- Pre-travel assessment: Individuals with prior VTE, known thrombophilia, or recent COVID-19 should consult a physician before high-altitude travel.

## 8 Conclusion

High altitude is an independent risk factor for venous thromboembolism, mediated by a complex pathophysiological network centered on HIF-driven multi-system disruption of hemostatic homeostasis. Current VTE risk tools and guidelines, designed for low-altitude populations, lack sensitivity in high-altitude environments. We propose a three-dimensional (altitude–exposure–susceptibility) risk assessment framework to enable precision prevention. Critical evidence gaps include the absence of altitude-adjusted D-dimer cutoffs, uncharacterized anticoagulant pharmacokinetics at high altitude, and a lack of randomized controlled trials in rapid ascenders. Future research should prioritize multi-omics discovery, novel animal models, and rigorously designed clinical trials to shift from population-based to precision prevention of high-altitude VTE.
